# Supplementary material for: Inference of interactions between chromatin modifiers and histone modifications: from ChIP-Seq data to chromatin-signaling
Source: Nucleic Acids Res. 2014 Nov 20;42(22):13689–95. doi: 10.1093/nar/gku1234 (PMC4267652; doi:10.1093/nar/gku1234)
Supplement: SUPPLEMENTARY DATA [file supp_42_22_13689__index.html]

Inference of interactions between chromatin modifiers and histone modifications: from ChIP-Seq data to chromatin-signaling — Inference of interactions between chromatin modifiers and histone modifications: from ChIP-Seq data to chromatin-signaling — SUPPLEMENTARY DATA 

# Inference of interactions between chromatin modifiers and histone modifications: from ChIP-Seq data to chromatin-signaling

## SUPPLEMENTARY DATA

**Files in this Data Supplement:**

- SUPPLEMENTARY DATA
