# Supplementary material for: Inference of interactions between chromatin modifiers and histone modifications: from ChIP-Seq data to chromatin-signaling
Source: Nucleic Acids Res. 2014 Nov 20;42(22):13689–95. doi: 10.1093/nar/gku1234 (PMC4267652; doi:10.1093/nar/gku1234)
Supplement: SUPPLEMENTARY DATA [file supp_gku1234_nar-02941-n-2014-File007.pdf]

Supplementary Note S1

Many of the interactions in the network in Figure 3 (main text) are supported by published experimental evidence. Here we discuss the experimental evidence for these interactions in more detail. The discussion for the H3K27me3-related edges is given in the main text.

H3K36me3 is positively associated to WHSC1, suggesting that WHSC1 either binds or catalyzes H3K36me3. Biochemical evidence suggests that WHSC1 methylates H3K36 (1, 2), supporting the detected positive association. The positive associations between Pol II and H3K36me3, H3K79me2 and H4K20me1 indicate that Pol II is involved in either binding or catalyzing these HMs. Indeed, Pol II associates with SETD2, which sets H3K36me3 (3), DOT1L, which sets H3K79me2 (4) and SETD8, which sets H4K20me1 (5). Although, these interactions of the HMs to Pol II are not direct because they involve additional proteins, here they are identified as direct due to the lack of ChIP-Seq data accounting for SETD2, DO1L and SETD8 in the present data set.

Apart from CBX2 and CBX8, which directly bind H3K27me3, we uncover one additional example where biochemical experiments show that a CM binds directly to an HM, namely CBX3 and H3K9me3 (6, 7), which is supported by a positive interaction in our network. Another example of such a direct interaction is the negative link between WHSC1 and H3K9me3, which is supported by the observation that WHSC1 binding is inhibited by the presence of H3K9me3 (8). The negative interaction between SETDB1 and H3K4me1 suggests that SETDB1 either drives the steady state equilibrium away from H3K4me1, or that its chromatin association is inhibited by the presence of H3K4me1. SETDB1 is a histone methyltransferase for H3K9 (9), rendering an interference with the steady state levels of H3K4me1 unlikely. Moreover, it has been shown that SETDB1 binding is inhibited by H3K4me3 (10), which may also be the case for H3K4me1. Consistently, H3K4me1 is often found at open chromatin regions, e.g. enhancers (11), whereas SETDB1 is associated with heterochromatin (9).

The positive association between WHSC1 and H3K4me1 suggests that WHSC1 may be involved in increasing the steady state levels of H3K4me1, or that H3K4me1 promotes the binding of WHSC1 to chromatin. WHSC1 harbors a SET-domain capable of methylating H4K20 (12) and H3K36 (1, 2), indicating that WHSC1 is unlikely to be involved in the metabolism of H3K4me1. Apart from the SET-domain, WHSC1 also contains a PHD5-C5HCH domain that has been shown to bind to unmodified H3K4 (8). The positive association between WHSC1 and H3K4me1 may indicate direct binding. Alternatively, WHSC1 could independently of its ability to bind to unmethylated H3K4 be part of a complex that is associated to H3K4me1. In line, WHSC1 interacts with the NuRD and the SIN3A complex and this interaction depends on the N-terminal tail of WHSC1 (12). There is one example, where a CM has been shown to remove an HM, i.e. KDM1A and H3K4me1 (13, 14). However, the uncovered relationship in our network is positive and thus may indicate the presence of KDM1A at H3K4me1 bearing loci. This could reflect that KDM1A cannot exert its demethylase activity or that it is involved in a constant turnover of H3K4me1, similar to the case of KDM5B and H3K4me3 (see below). The demethylase activity of KDM1A is increased by the presence of H3K9ac

(15), suggesting that the lack of H3K9ac at loci with high H3K4me1 may shift the equilibrium towards H3K4me1.

KDM5B is negatively associated to H3K36me3, suggesting that KDM5B either lowers the levels of H3K36me3, or that its chromatin association is reduced in the presence of H3K36me3. KDM5B is a H3K4me3-specific demethylase (16, 17) and modulates H3K4me3-levels at the TSS in murine embryonic stem cells (mESCs; (18). Furthermore, KDM5B has been proposed to associate with H3K36me3 in the gene body via MORF4L1 to restrict H3K4me3 to the promoter in mESCs (19), suggesting that H3K36me3 promotes rather than inhibits binding of KDM5B. However, it has been shown that the KDM5B antibody used enriches intragenic regions even in KDM5B knockout ESCs, arguing that the gene body enrichment by this antibody is not specific for KDM5B (18). Experimental data does not provide any mechanistic explanation for the negative interaction between KDM5B and H3K36me3. However, our analysis suggests that this interaction is not mediated by targets of KDM5B H3K4me1/2/3, because we recover the link even after accounting for H3K4me1/2/3 in the partial correlation framework. Based on this line of reasoning KDM5B may be involved in demethylating H3K36me3 or its chromatin association is inhibited by the presence of H3K36me3.

Supplementary Figure S1: Reproducibility of ChIP-seq data

The barplot shows the coefficient of determination (R^2) of the HM levels at promoters between biological replicates.

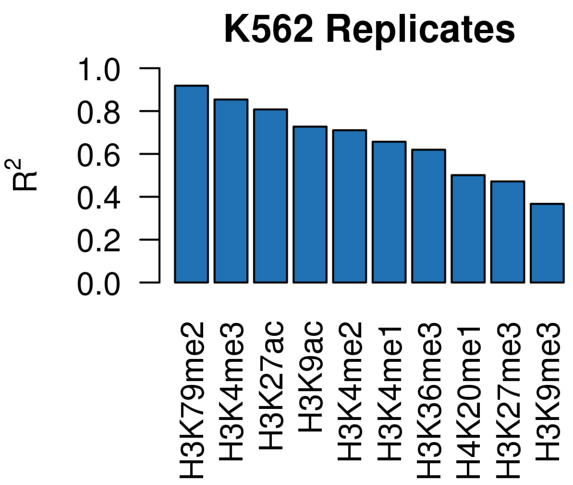

Supplementary Figure S2: Pairwise correlations

The heatmap shows the raw pairwise correlation matrix. Each entry gives the color coded Pearson correlation coefficient between two variables as indicated in the color key. The data is reordered to show groups of HMs and CMs with similar correlation profiles. The entries corresponding to self-correlation have been removed.

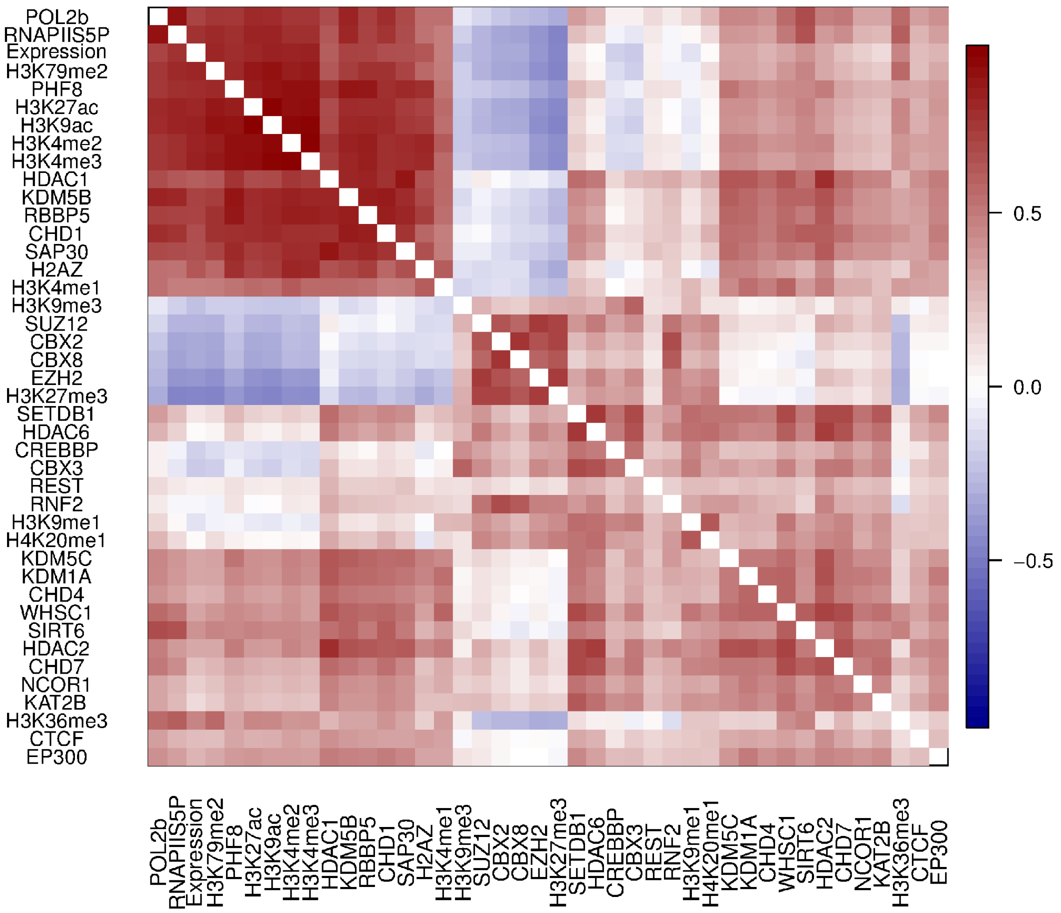

Supplementary Figure S3: Chromatin-signaling networks

A graphical representation of the interactions between CMs (circles) and HMs (squares) detected in (**A**) the Elastic Net and (**B**) the SPCN. The arrows on the links in (**A**) only represent the design of the linear model and should not be interpreted as causal relationships. Red lines indicate positive, blue lines negative interactions.

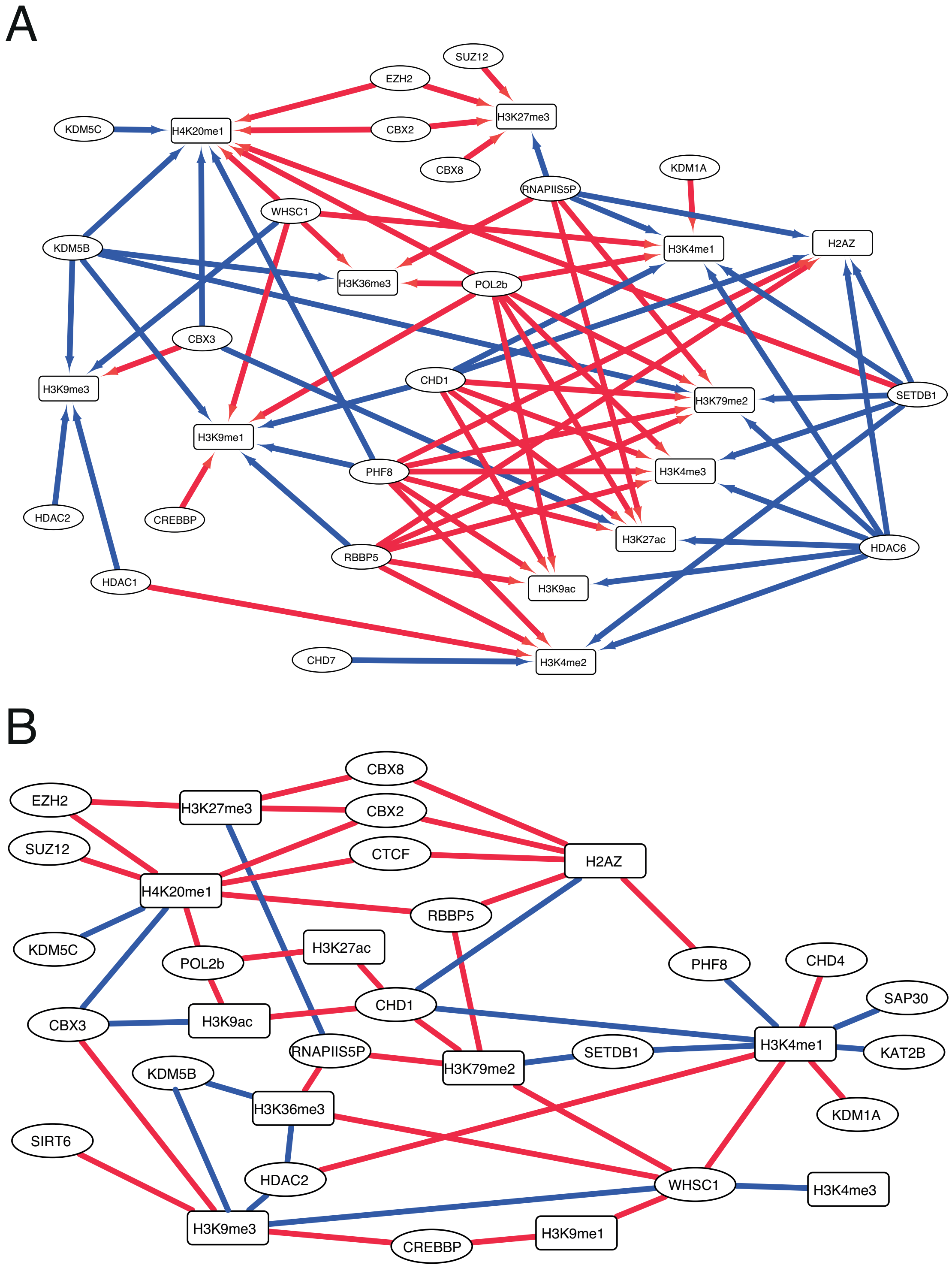

Supplementary Figure S4: The regression coefficients of the CMs used to predict the HM levels

In each boxplot, the regression coefficients of the CMs used to predict an HM by Elastic Net in 10-fold cross-validation are depicted. For each CM, the boxes indicate the upper and lower quartile, the bold line indicates the median, and the upper and lower whiskers indicate the minimum and maximum of all CV-coefficients. The dashed line corresponds to the average of all coefficients and the two dash-dot lines correspond to ± one standard deviation of all coefficients.

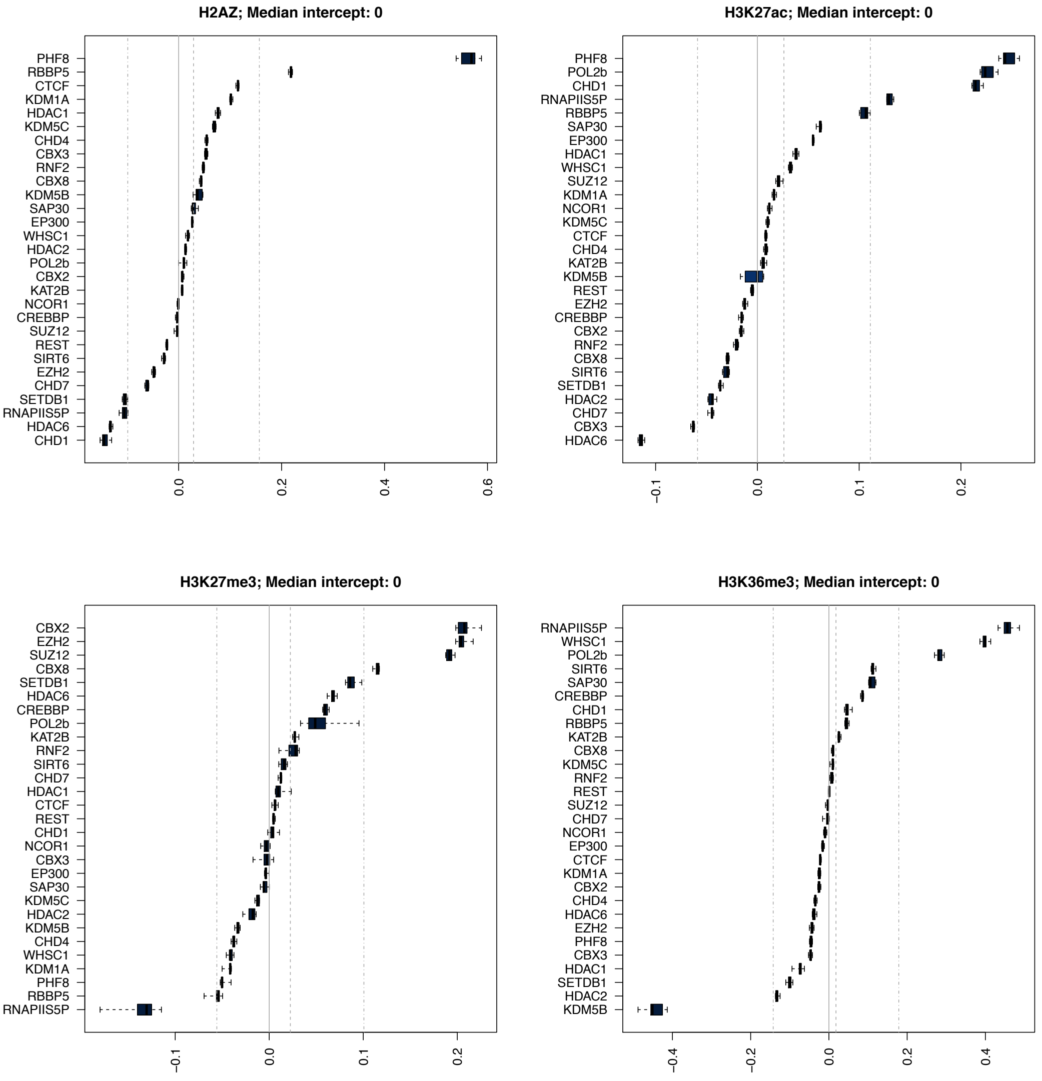

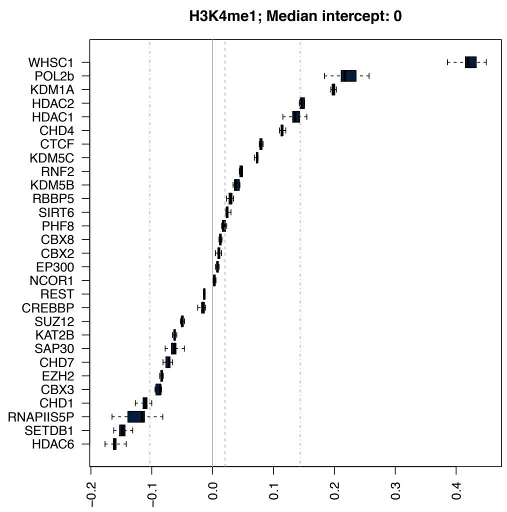
H3K4me1; Median intercept: 0
WHSC1
POL2b
KDM1A
HDAC2
HDAC1
CHD4
CTCF
KDM5C
RNF2
KDM5B
RBBP5
SIRT6
PHF8
CBX8
CBX2
EP300
NCOR1
REST
CREBBP
SUZ12
KAT2B
SAP30
CHD7
EZH2
CBX3
CHD1
RNAPIIS5P
SETDB1
HDAC6
−0.2
−0.1
0.0
0.1
0.2
0.3
0.4

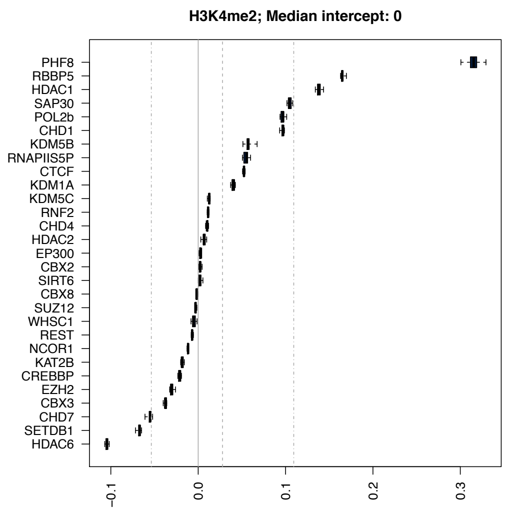
H3K4me2; Median intercept: 0
PHF8
RBBP5
HDAC1
SAP30
POL2b
CHD1
KDM5B
RNAPIIS5P
CTCF
KDM1A
KDM5C
RNF2
CHD4
HDAC2
EP300
CBX2
SIRT6
CBX8
SUZ12
WHSC1
REST
NCOR1
KAT2B
CREBBP
EZH2
CBX3
CHD7
SETDB1
HDAC6
−0.1
0.0
0.1
0.2
0.3

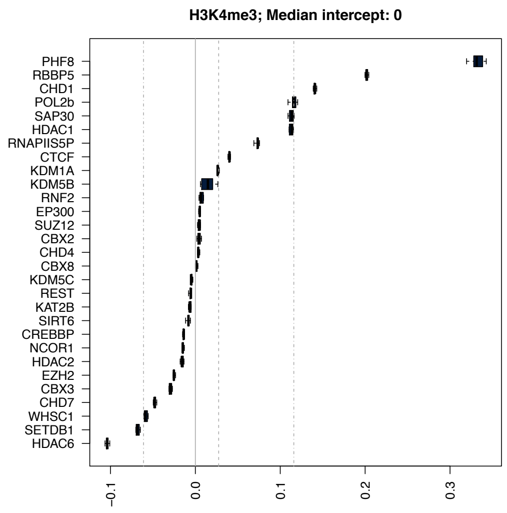
H3K4me3; Median intercept: 0
PHF8
RBBP5
CHD1
POL2b
SAP30
HDAC1
RNAPIIS5P
CTCF
KDM1A
KDM5B
RNF2
EP300
SUZ12
CBX2
CHD4
CBX8
KDM5C
REST
KAT2B
SIRT6
CREBBP
NCOR1
HDAC2
EZH2
CBX3
CHD7
WHSC1
SETDB1
HDAC6
−0.1
0.0
0.1
0.2
0.3

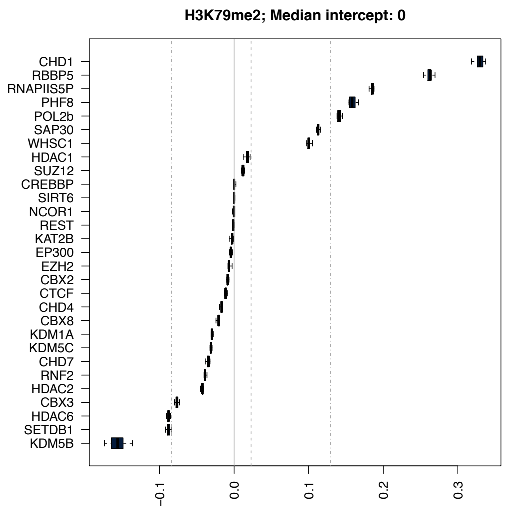
H3K79me2; Median intercept: 0
CHD1
RBBP5
RNAPIIS5P
PHF8
POL2b
SAP30
WHSC1
HDAC1
SUZ12
CREBBP
SIRT6
NCOR1
REST
KAT2B
EP300
EZH2
CBX2
CTCF
CHD4
CBX8
KDM1A
KDM5C
CHD7
RNF2
HDAC2
CBX3
HDAC6
SETDB1
KDM5B
−0.1
0.0
0.1
0.2
0.3

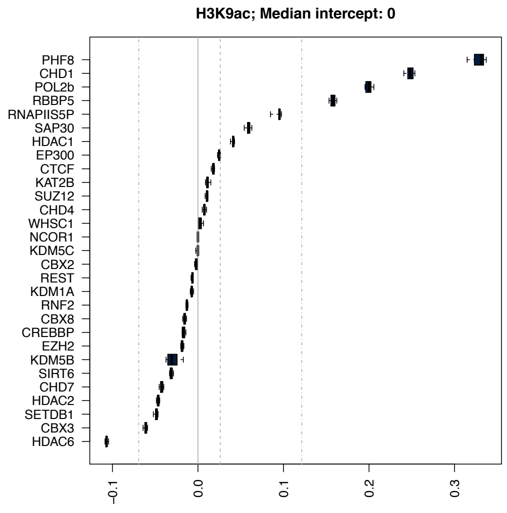
H3K9ac; Median intercept: 0
PHF8
CHD1
POL2b
RBBP5
RNAPIIS5P
SAP30
HDAC1
EP300
CTCF
KAT2B
SUZ12
CHD4
WHSC1
NCOR1
KDM5C
CBX2
REST
KDM1A
RNF2
CBX8
CREBBP
EZH2
KDM5B
SIRT6
CHD7
HDAC2
SETDB1
CBX3
HDAC6
−0.1
0.0
0.1
0.2
0.3

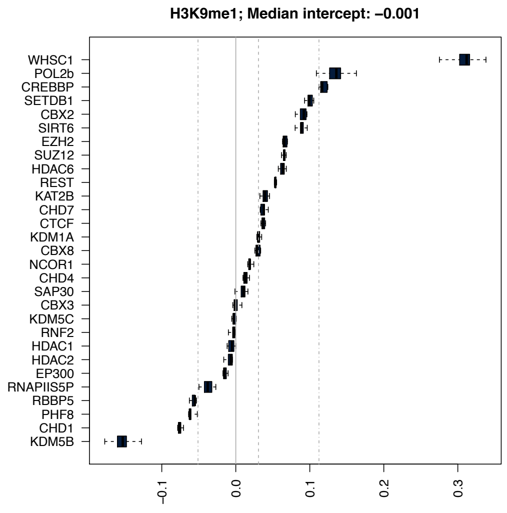
H3K9me1; Median intercept: −0.001
WHSC1
POL2b
CREBBP
SETDB1
CBX2
SIRT6
EZH2
SUZ12
HDAC6
REST
KAT2B
CHD7
CTCF
KDM1A
CBX8
NCOR1
CHD4
SAP30
CBX3
KDM5C
RNF2
HDAC1
HDAC2
EP300
RNAPIIS5P
RBBP5
PHF8
CHD1
KDM5B
−0.1
0.0
0.1
0.2
0.3

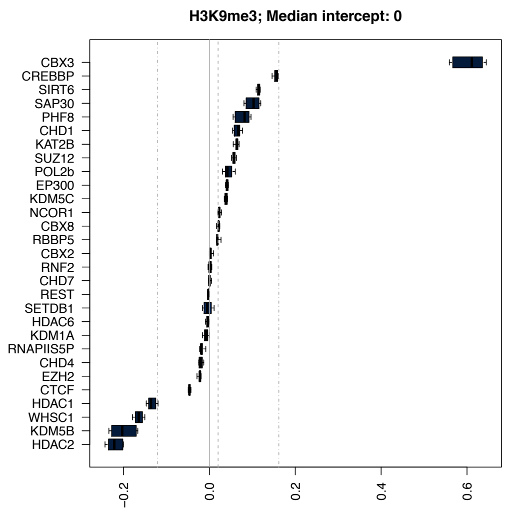
H3K9me3; Median intercept: 0
CBX3
CREBBP
SIRT6
SAP30
PHF8
CHD1
KAT2B
SUZ12
POL2b
EP300
KDM5C
NCOR1
CBX8
RBBP5
CBX2
RNF2
CHD7
REST
SETDB1
HDAC6
KDM1A
RNAPIIS5P
CHD4
EZH2
CTCF
HDAC1
WHSC1
KDM5B
HDAC2
−0.2
0.0
0.2
0.4
0.6

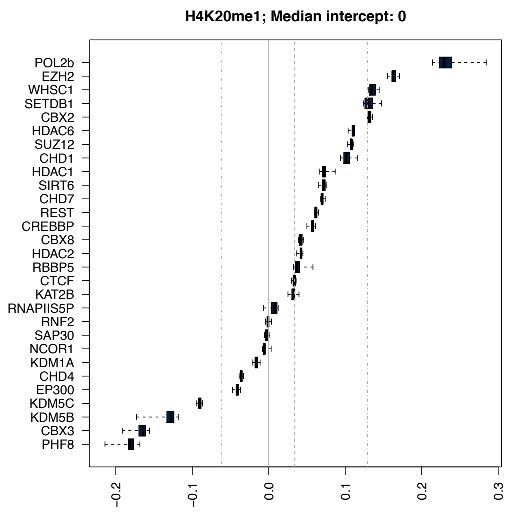
H4K20me1; Median intercept: 0
POL2b
EZH2
WHSC1
SETDB1
CBX2
HDAC6
SUZ12
CHD1
HDAC1
SIRT6
CHD7
REST
CREBBP
CBX8
HDAC2
RBBP5
CTCF
KAT2B
RNAPIIS5P
RNF2
SAP30
NCOR1
KDM1A
CHD4
EP300
KDM5C
KDM5B
CBX3
PHF8
−0.2
−0.1
0.0
0.1
0.2
0.3

Supplementary Figure S5: Pairwise partial correlations

The heatmap shows the partial correlation matrix where each entry gives the correlation between two variables controlled for all other variables. The entries corresponding to self-correlation have been removed. The boxes indicate manually derived groups of correlated variables.

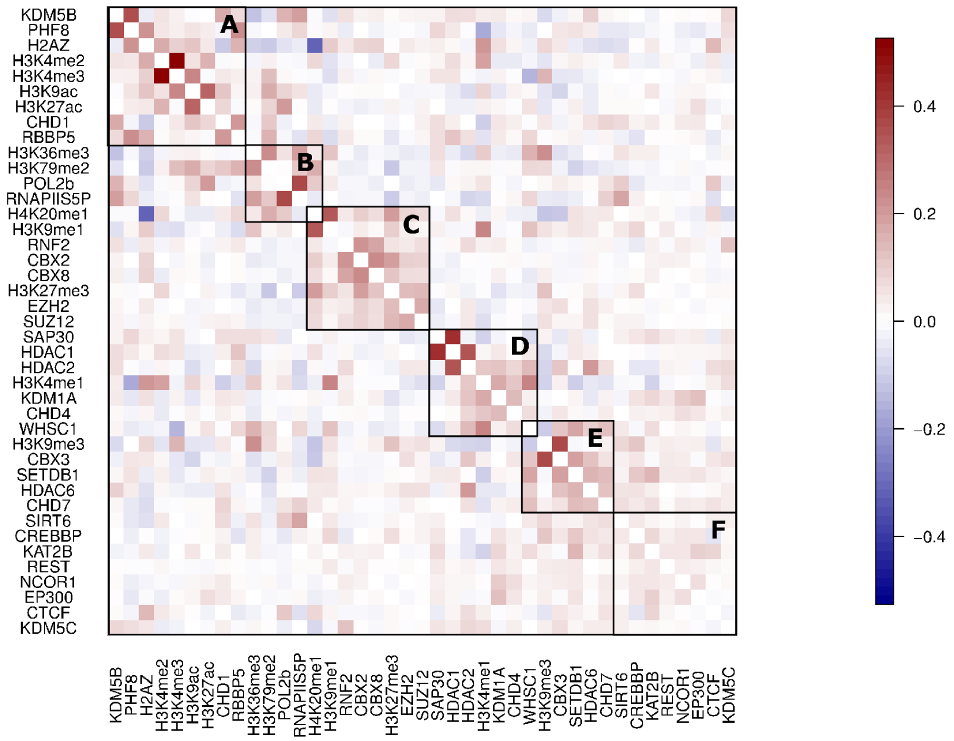

Supplementary Table S1: Known interactions between histone residues and chromatin modifiers

The table lists examples of CMs in the data set that are known to interact positively or negatively with the methylation or acetylation status of the listed residues.

Residue	CM	Ref.
**H3K4**	KDM1A	(13, 14)
**H3K4**	KDM5A	(20)
**H3K4**	KDM5B	(17)
**H3K4**	KDM5C	(21),
**H3K4**	CHD1	(22, 23)
**H3K4**	PHF8	(24-27)
**H3K4**	RBBP5	(28)
**H3K9**	SETDB1	(9)
**H3K9**	CBX3	(6, 7)
**H3K9**	PHF8	(29)
**H3K27**	SUZ12	(30, 31)
**H3K27**	EZH2	(32-35)
**H3K27**	CBX2	(34)
**H3K27**	CBX8	(34)
**H3K27**	PHF8	(29)

Supplementary Table S2: Summary of evidence for each association in the networks

Each interaction can be positive (1) or negative (-1) in each network (column "EN" and "SPCN"). The literature evidence (column "reference") can be indirect via maximal one other protein for which ChIP-Seq data was not available.

HM	CM	EN	SPCN	Reference	Comment
H2A.Z	CHD1	-1	-1	(36)	CHD1 is involved in the removal of H2A.Z in the gene body
H2A.Z	PHF8	1	1		
H2A.Z	RBBP5	1	1	(37)	H2A.Z recruits RBBP5
H3K27ac	CHD1	1	1		
H3K27ac	Pol2b	1	1	(38, 39)	Elongator complex carries HAT activity, Indicator of active promoter
H3K27me3	CBX2	1	1	(34)	CBX2 binds to H3K27me3
H3K27me3	CBX8	1	1	(34)	CBX8 binds to H3K27me3
H3K27me3	EZH2	1	1	(32-35)	EZH2 catalyzes H3K27me3
H3K27me3	SUZ12	1	1	(31, 40)	SUZ12 binds H3K27me3 via EED
H3K27me3	RNAPIIS5P	-1	-1	(41)	High levels of RNAPIIS5P indicate elongation, which is repressed by H3K27me3
H3K36me3	KDM5B	-1	-1		
H3K36me3	RNAPIIS5P	1	1	(3)	Pol II carries SETD2 which methylates H3K36
H3K36me3	WHSC1	1	1	(1, 2)	WHSC1 methylates H3K36
H3K4me1	CHD1	-1	-1		
H3K4me1	KDM1A	1	1	(13, 14)	KDM1A demethylates H3K4me1
H3K4me1	SETDB1	-1	-1	(10)	SETDB1 binds less well to H3K4me1 substrates
H3K4me1	WHSC1	1	1	(8)	WHSC1 binds to H3K4 unmodified
H3K79me2	CHD1	1	1		
H3K79me2	RBBP5	1	1	(4, 42)	May reflect the effect of RBBP5-mediated H2BK120ub, that boosts DOT1L activity
H3K79me2	RNAPIIS5P	1	1	(4)	Pol II carries DOT1L which methylates H3K79
H3K79me2	SETDB1	-1	-1		
H3K9ac	CHD1	1	1		
H3K9ac	Pol2b	1	1	(38, 39)	Elongator complex carries HAT activity, Indicator of active promoter
H3K9me1	CREBBP	1	1		
H3K9me1	WHSC1	1	1		
H3K9me3	CBX3	1	1	(6, 7)	CBX3 binds to H3K9me3
H3K9me3	HDAC2	-1	-1		
H3K9me3	KDM5B	-1	-1		
H3K9me3	WHSC1	1	1	(8)	WHSC1 binds less well to H3K9me3 substrates
H4K20me1	CBX2	1	1	This study	
H4K20me1	CBX3	-1	-1		
H4K20me1	EZH2	1	1	This study	
H4K20me1	KDM5C	-1	-1		
H4K20me1	Pol2b	1	1	(5)	Pol II carries SETD8 which methylates H4K20

Supplementary References

1. Li, Y., Trojer, P., Xu, C.-F., Cheung, P., Kuo, A., Drury, W.J., Qiao, Q., Neubert, T.A., Xu, R.-M., Gozani, O., et al. (2009) The target of the NSD family of histone lysine methyltransferases depends on the nature of the substrate. *J. Biol. Chem.*, **284**, 34283–34295.

2. Nimura, K., Ura, K., Shiratori, H., Ikawa, M., Okabe, M., Schwartz, R.J. and Kaneda, Y. (2009) A histone H3 lysine 36 trimethyltransferase links Nkx2-5 to Wolf-Hirschhorn syndrome. *Nature*, **460**, 287–291.

3. Kizer, K.O., Phatnani, H.P., Shibata, Y., Hall, H., Greenleaf, A.L. and Strahl, B.D. (2005) A novel domain in Set2 mediates RNA polymerase II interaction and couples histone H3 K36 methylation with transcript elongation. *Mol. Cell. Biol.*, **25**, 3305–3316.

4. Kim, S.-K., Jung, I., Lee, H., Kang, K., Kim, M., Jeong, K., Kwon, C.S., Han, Y.-M., Kim, Y.S., Kim, D., et al. (2012) Human histone H3K79 methyltransferase DOT1L methyltransferase binds actively transcribing RNA polymerase II to regulate gene expression. *J. Biol. Chem.*, **287**, 39698–39709.

5. Li, Y., Sun, L., Zhang, Y., Wang, D., Wang, F., Liang, J., Gui, B. and Shang, Y. (2011) The histone modifications governing TFF1 transcription mediated by estrogen receptor. *J. Biol. Chem.*, **286**, 13925–13936.

6. Lachner, M., O'Carroll, D., Rea, S., Mechtler, K. and Jenuwein, T. (2001) Methylation of histone H3 lysine 9 creates a binding site for HP1 proteins. *Nature*, **410**, 116–120.

7. Bannister, A.J., Zegerman, P., Partridge, J.F., Miska, E.A., Thomas, J.O., Allshire, R.C. and Kouzarides, T. (2001) Selective recognition of methylated lysine 9 on histone H3 by the HP1 chromo domain. *Nature*, **410**, 120–124.

8. He, C., Li, F., Zhang, J., Wu, J. and Shi, Y. (2013) The Methyltransferase NSD3 Has Chromatin-binding Motifs, PHD5-C5HCH, That Are Distinct from Other NSD (Nuclear Receptor SET Domain) Family Members in Their Histone H3 Recognition. *J. Biol. Chem.*

9. Schultz, D.C., Ayyanathan, K., Negorev, D., Maul, G.G. and Rauscher, F.J. (2002) SETDB1: a novel KAP-1-associated histone H3, lysine 9-specific methyltransferase that contributes to HP1-mediated silencing of euchromatic genes by KRAB zinc-finger proteins. *Genes Dev.*, **16**, 919–932.

10. Binda, O., Leroy, G., Bua, D.J., Garcia, B.A., Gozani, O. and Richard, S. (2010) Trimethylation of histone H3 lysine 4 impairs methylation of histone H3 lysine 9: regulation of lysine methyltransferases by physical interaction with their substrates. *Epigenetics*, **5**, 767–775.

11. Heintzman, N.D., Stuart, R.K., Hon, G., Fu, Y., Ching, C.W., Hawkins, R.D., Barrera, L.O., Van Calcar, S., Qu, C., Ching, K.A., et al. (2007) Distinct and predictive chromatin signatures of transcriptional promoters and enhancers in the human genome. *Nat. Genet.*, **39**, 311–318.

12. Marango, J., Shimoyama, M., Nishio, H., Meyer, J.A., Min, D.-J., Sirulnik, A., Martinez-Martinez, Y., Chesi, M., Bergsagel, P.L., Zhou, M.-M., et al. (2008) The MMSET protein is a histone methyltransferase with characteristics of a transcriptional corepressor. *Blood*, **111**, 3145–3154.

13. Forneris, F., Binda, C., Vanoni, M.A., Mattevi, A. and Battaglioli, E. (2005) Histone demethylation catalysed by LSD1 is a flavin-dependent oxidative process. *FEBS Lett.*, **579**, 2203–2207.

14. Shi, Y., Lan, F., Matson, C., Mulligan, P., Whetstine, J.R., Cole, P.A., Casero, R.A. and Shi, Y. (2004) Histone demethylation mediated by the nuclear amine oxidase homolog LSD1. *Cell*, **119**, 941–953.

15. Forneris, F., Binda, C., Vanoni, M.A., Battaglioli, E. and Mattevi, A. (2005) Human histone demethylase LSD1 reads the histone code. *J. Biol. Chem.*, **280**, 41360–41365.

16. Seward, D.J., Cubberley, G., Kim, S., Schonewald, M., Zhang, L., Tripet, B. and Bentley, D.L. (2007) Demethylation of trimethylated histone H3 Lys4 in vivo by JARID1 JmjC proteins. *Nat. Struct. Mol. Biol.*, **14**, 240–242.

17. Yamane, K., Tateishi, K., Klose, R.J., Fang, J., Fabrizio, L.A., Erdjument-Bromage, H., Taylor-Papadimitriou, J., Tempst, P. and Zhang, Y. (2007) PLU-1 is an H3K4 demethylase involved in transcriptional repression and breast cancer cell proliferation. *Mol. Cell*, **25**, 801–812.

18. Schmitz, S.U., Albert, M., Malatesta, M., Morey, L., Johansen, J.V., Bak, M., Tommerup, N., Abarrategui, I. and Helin, K. (2011) Jarid1b targets genes regulating development and is involved in neural differentiation. *EMBO J.*, **30**, 4586–4600.

19. Xie, L., Pelz, C., Wang, W., Bashar, A., Varlamova, O., Shadle, S. and Impey, S. (2011) KDM5B regulates embryonic stem cell self-renewal and represses cryptic intragenic transcription. *EMBO J.*, **30**, 1473–1484.

20. Hayakawa, T., Ohtani, Y., Hayakawa, N., Shinmyozu, K., Saito, M., Ishikawa, F. and Nakayama, J.-I. (2007) RBP2 is an MRG15 complex component and down-regulates intragenic histone H3 lysine 4 methylation. *Genes Cells*, **12**, 811–826.

21. Iwase, S., Lan, F., Bayliss, P., la Torre-Ubieta, de, L., Huarte, M., Qi, H.H., Whetstine, J.R., Bonni, A., Roberts, T.M. and Shi, Y. (2007) The X-linked mental retardation gene SMCX/JARID1C defines a family of histone H3 lysine 4 demethylases. *Cell*, **128**, 1077–1088.

22. Sims, R.J., Chen, C.-F., Santos-Rosa, H., Kouzarides, T., Patel, S.S. and Reinberg, D. (2005) Human but not yeast CHD1 binds directly and selectively to histone H3 methylated at lysine 4 via its tandem chromodomains. *J. Biol. Chem.*, **280**, 41789–41792.

23. Pray-Grant, M.G., Daniel, J.A., Schieltz, D., Yates, J.R. and Grant, P.A. (2005) Chd1 chromodomain links histone H3 methylation with SAGA- and SLIK-dependent acetylation. *Nature*, **433**, 434–438.

24. Vermeulen, M., Eberl, H.C., Matarese, F., Marks, H., Denissov, S., Butter, F., Lee, K.K., Olsen, J.V., Hyman, A.A., Stunnenberg, H.G., et al. (2010) Quantitative interaction proteomics and genome-wide profiling of epigenetic histone marks and their readers. *Cell*, **142**, 967–980.

25. Horton, J.R., Upadhyay, A.K., Qi, H.H., Zhang, X., Shi, Y. and Cheng, X. (2010) Enzymatic and structural insights for substrate specificity of a family of jumonji histone lysine demethylases. *Nat. Struct. Mol. Biol.*, **17**, 38–43.

26. Fortschegger, K., de Graaf, P., Outchkourov, N.S., van Schaik, F.M.A., Timmers, H.T.M. and Shiekhattar, R. (2010) PHF8 targets histone methylation and RNA polymerase II to activate transcription. *Mol. Cell. Biol.*, **30**, 3286–3298.

27. Feng, W., Yonezawa, M., Ye, J., Jenuwein, T. and Grummt, I. (2010) PHF8 activates transcription of rRNA genes through H3K4me3 binding and H3K9me1/2 demethylation. *Nat. Struct. Mol. Biol.*, **17**, 445–450.

28. Shilatifard, A. (2012) The COMPASS family of histone H3K4 methylases: mechanisms of regulation in development and disease pathogenesis. *Annu. Rev. Biochem.*, **81**, 65–95.

29. Qi, H.H., Sarkissian, M., Hu, G.-Q., Wang, Z., Bhattacharjee, A., Gordon, D.B., Gonzales, M., Lan, F., Ongusaha, P.P., Huarte, M., et al. (2010) Histone H4K20/H3K9 demethylase PHF8 regulates zebrafish brain and craniofacial development. *Nature*, **466**, 503–507.

30. Cao, R. and Zhang, Y. (2004) SUZ12 is required for both the histone methyltransferase activity and the silencing function of the EED-EZH2 complex. *Mol. Cell*, **15**, 57–67.

31. Hansen, K.H., Bracken, A.P., Pasini, D., Dietrich, N., Gehani, S.S., Monrad, A., Rappsilber, J., Lerdrup, M. and Helin, K. (2008) A model for transmission of the H3K27me3 epigenetic mark. *Nat. Cell Biol.*, **10**, 1291–1300.

32. Müller, J., Hart, C.M., Francis, N.J., Vargas, M.L., Sengupta, A., Wild, B., Miller, E.L., O'Connor, M.B., Kingston, R.E. and Simon, J.A. (2002) Histone methyltransferase activity of a Drosophila Polycomb group repressor complex. *Cell*, **111**, 197–208.

33. Kuzmichev, A., Nishioka, K., Erdjument-Bromage, H., Tempst, P. and Reinberg, D. (2002) Histone methyltransferase activity associated with a human multiprotein complex containing the Enhancer of Zeste protein. *Genes Dev.*, **16**, 2893–2905.

34. Cao, R., Wang, L., Wang, H., Xia, L., Erdjument-Bromage, H., Tempst, P., Jones, R.S. and Zhang, Y. (2002) Role of histone H3 lysine 27 methylation in Polycomb-group silencing. *Science*, **298**, 1039–1043.

35. Czermin, B., Melfi, R., McCabe, D., Seitz, V., Imhof, A. and Pirrotta, V. (2002) Drosophila enhancer of Zeste/ESC complexes have a histone H3 methyltransferase activity that marks chromosomal Polycomb sites. *Cell*, **111**, 185–196.

36. Persson, J. and Ekwall, K. (2010) Chd1 remodelers maintain open chromatin and regulate the epigenetics of differentiation. *Exp. Cell Res.*, **316**, 1316–1323.

37. Hu, G., Cui, K., Northrup, D., Liu, C., Wang, C., Tang, Q., Ge, K., Levens, D., Crane-Robinson, C. and Zhao, K. (2013) H2A.Z Facilitates Access of Active and Repressive Complexes to Chromatin in Embryonic Stem Cell Self-Renewal and Differentiation. *Cell Stem Cell*, **12**, 180–192.

38. Wittschieben, B.O., Otero, G., de Bizemont, T., Fellows, J., Erdjument-Bromage, H., Ohba, R., Li, Y., Allis, C.D., Tempst, P. and Svejstrup, J.Q. (1999) A novel histone acetyltransferase is an integral subunit of elongating RNA polymerase II holoenzyme. *Mol. Cell*, **4**, 123–128.

39. Kim, J.-H., Lane, W.S. and Reinberg, D. (2002) Human Elongator facilitates RNA

polymerase II transcription through chromatin. *Proc. Natl. Acad. Sci. U.S.A.*, **99**, 1241–1246.

40. Margueron, R., Justin, N., Ohno, K., Sharpe, M.L., Son, J., Drury, W.J., Voigt, P., Martin, S.R., Taylor, W.R., De Marco, V., et al. (2009) Role of the polycomb protein EED in the propagation of repressive histone marks. *Nature*, **461**, 762–767.

41. Bataille, A.R., Jeronimo, C., Jacques, P.-É., Laramée, L., Fortin, M.-È., Forest, A., Bergeron, M., Hanes, S.D. and Robert, F. (2012) A Universal RNA Polymerase II CTD Cycle Is Orchestrated by Complex Interplays between Kinase, Phosphatase, and Isomerase Enzymes along Genes. *Mol. Cell*, **45**, 158–170.

42. Frederiks, F., Tzouros, M., Oudgenoeg, G., van Welsem, T., Fornerod, M., Krijgsveld, J. and van Leeuwen, F. (2008) Nonprocessive methylation by Dot1 leads to functional redundancy of histone H3K79 methylation states. *Nat. Struct. Mol. Biol.*, **15**, 550–557.
